# Health-system resilience: reflections on the Ebola crisis in western Africa

**DOI:** 10.2471/BLT.14.149278

**Published:** 2014-12-01

**Authors:** Marie-Paule Kieny, David B Evans, Gerard Schmets, Sowmya Kadandale

**Affiliations:** a World Health Organization, avenue Appia 20, 1211 Geneva 27, Switzerland.

Disease outbreaks and catastrophes can affect countries at any time, causing substantial human suffering and deaths and economic losses. If health systems are ill-equipped to deal with such situations, the affected populations can be very vulnerable.^1^

The current Ebola virus disease outbreak in western Africa highlights how an epidemic can proliferate rapidly and pose huge problems in the absence of a strong health system capable of a rapid and integrated response. The outbreak began in Guinea in December 2013 but soon spread into neighbouring Liberia and Sierra Leone.^2^ In early August 2014, Ebola was declared an international public health emergency.^2^

At the time the outbreak began, the capacity of the health systems in Guinea, Liberia and Sierra Leone was limited. Several health-system functions that are generally considered essential were not performing well and this hampered the development of a suitable and timely response to the outbreak. There were inadequate numbers of qualified health workers.^3^ Infrastructure, logistics, health information, surveillance, governance and drug supply systems were weak. The organization and management of health services was sub-optimal. Government health expenditure was low whereas private expenditure – mostly in the form of direct out-of-pocket payments for health services – was relatively high.^4^

The last decade has seen increased external health-related aid to Guinea, Liberia and Sierra Leone. However, in the context of Millennium Development Goals 4, 5 and 6, most of this aid has been allocated to combat human immunodeficiency virus infection, malaria and tuberculosis, with much of the residual going to maternal and child health services. Therefore, relatively little external aid was left to support overall development of health systems.^5^ This lack of balanced investment in the health systems contributes to the challenges of controlling the current Ebola outbreak. Weak health systems cannot be resilient.^6^^–^^8^ A strong health system decreases a country’s vulnerability to health risks and ensures a high level of preparedness to mitigate the impact of any crises.

Frequently, the response by governments and external partners to a health crisis posed by a communicable disease, such as Ebola, is to focus solely on reducing transmission and the effect of the disease. However, such a response is insufficient. Febrile individuals need to be screened for Ebola – even if most of them have fevers caused by other infections – and those found to be negative for Ebola still need to be treated rather than simply turned away. Even in the worst-affected areas, women still need antenatal services, safe delivery and postnatal care. Many people will travel to seek care for unrelated conditions in areas that they perceive to be Ebola-free, putting enormous strain on the health system in so-called “non-Ebola” areas. Routine services need to be assured while dealing with the direct effects of an epidemic. Otherwise, more people may die – of unrelated causes – from a general breakdown of health services than as a direct result of the epidemic.

If this Ebola outbreak does not trigger substantial investments in health systems and adequate reforms in the worst-affected countries, pre-existing deficiencies in health systems will be exacerbated. The national governments, assisted by external partners, need to develop and implement strategies to make their health systems stronger and more resilient. Only then can they meet the essential health needs of their populations and develop strong disaster preparedness to address future emergencies. In the short-term, nongovernmental organizations, civil society and international organizations will have to bolster the national health systems, both to mitigate the direct consequences of the outbreak and to ensure that all essential health services are being delivered. However, this assistance should be carefully coordinated under the leadership of the national governments and follow development effectiveness principles. We expect health systems in the worst-affected areas to be left in a very weak state once the outbreak has ended. Hopefully, after the epidemic has ended, economic growth and government health spending will eventually rebound, with increased domestic investments in health systems. For the foreseeable future however, the negative economic impact on the affected countries^9^ means that substantial external financing will be needed to build stronger national and subnational health systems.
